# Evaluation of Pain and Opioid Use in Ptosis and Blepharoplasty Surgery

**DOI:** 10.1155/joph/7910156

**Published:** 2026-04-24

**Authors:** Christopher Bair, Bhupendra C. K. Patel

**Affiliations:** ^1^ Department of Ophthalmology, University of Utah, Salt Lake City, Utah, USA, utah.edu

**Keywords:** blepharoplasty, opioid, postoperative pain, ptosis

## Abstract

**Purpose:**

The purpose of this study was to quantify the postoperative pain experience of patients undergoing ptosis repair and/or blepharoplasty and to determine the actual number of opioid pills used by patients with the goal of reducing postoperative opioid prescriptions.

**Methods:**

In Part I of the study, 40 consecutive patients undergoing eyelid surgery were asked to rate their pain levels at specified time points following surgery. In Part II of the study, each patient between November 2017 and January 2020 undergoing eyelid surgery was prescribed six narcotic pills. They were contacted at 2 weeks postsurgery and asked how many narcotic pills they actually used and whether they had used other non‐narcotic analgesic methods.

**Results:**

In Part I of the study, pain levels were higher in men and peaked at 6 h after surgery with an average rating of 3.8 and steadily decreased to an average of 1.1 at 7 days postprocedure. In Part II of the study, 286 patients (108 male and 178 female) were included. The mean number of narcotic pills used was 4.1, with male patients using slightly more pills than female (4.00 vs. 5.00, *p* < 0.001). 205 of 286 participants (72%) used a non‐narcotic analgesic, and 49 of 286 participants (17%) indicated use of marijuana.

**Conclusions:**

Blepharoplasty and ptosis repair have low levels of postoperative pain. Further, this study demonstrates that six opioid pills is a reasonable prescribing guideline and that opioid prescriptions can be safely reduced without compromising patient comfort and pain.

## 1. Introduction

The overuse and abuse of opioid medications has become an area of increasing concern in the United States: opioid abuse was declared a public health crisis by the Department of Health and Human Services in 2017 [1]. Data from the 2019 National Survey on Drug Use and Health estimates that 1.7 million Americans suffer from an opioid use disorder [2]. According to data from the Centers for Disease Control (CDC), nearly 841,000 people have died between 1999 and 2019 from a drug overdose, with over 70,000 deaths in the year 2019 alone [3]. Opioids have been a major driver of overdose death, with 72.9% of overdose deaths in 2019 due to opioid use. Unfortunately, the situation has only worsened during the COVID‐19 pandemic, with CDC data estimating a record 100,000 drug overdose deaths, an increase of 28.5% from the year before [4].

Blepharoplasty and ptosis repair are among the most common procedures performed by oculoplastic and plastic surgeons throughout the world. To date, there is a lack of evidence‐based guidelines to guide physicians on postoperative opioid prescribing. This fact was highlighted in a presentation given by Jay Bishoff, MD at the Moran Eye Center in October 2018, where he showed prescribing data from surgeons in the Intermountain Healthcare System, one of the largest healthcare systems in the United States (Bishoff JT, presented at Moran Eye Center, Salt Lake City, 2018). For eyelid surgeries, the mean number of postoperative opioids prescribed was 15 pills per patient with substantial variation between prescribers, with some providers prescribing over 100 narcotic pills for use in the postoperative period. This number is not based on any published guidelines, but rather on surgeon preference and experience. The lack of prescribing guidelines with wide variation and excessive dosing in opioid prescriptions is a known concern and has been the subject of previous discussion [5, 6].

There are few studies that have examined both the patient experience and quantified the need for opioid medication following eyelid surgery. The purpose of our study was to quantify the postoperative pain experience of patients undergoing ptosis repair and/or blepharoplasty, and to determine the actual number of opioid pills used by patients with the goal of reducing postoperative opioid prescriptions.

## 2. Methods

This study was approved by the Institutional Review Board of the University of Utah. Data from the study were HIPAA compliant, and research methods adhered to the tenets of the Declaration of Helsinki. Patients 18 years of age and older undergoing upper eyelid blepharoplasty and/or ptosis repair at the Moran Eye Center with a single surgeon (BCKP) were included. All patients had preoperative and immediate postoperative anesthetic injection with a mixture of 2% lidocaine with 1:200,000 epinephrine and Marcaine 0.5% with buffer. The anterior (external) approach with skin incision and closure with a 6‐0 plain gut suture was used. A detailed informational handout was read by all patients in the clinic, and they were asked to read it again prior to the surgery. All patients were given ice packs to use intermittently for two to 3 days following surgery.

In Part I of the study, the aim was to quantify patient‐reported pain following upper eyelid surgery. 40 consecutive adult patients over the age of 18 undergoing upper eyelid surgery with a single surgeon (BCKP) at the Moran Eye Center were given the standard Numerical Rating Scale (NRS) 10‐point numerical pain scale and asked to rate their pain at predetermined timepoints after surgery. Each patient was made familiar with the pain scale and how to grade their pain [7]. Patients were excluded if they used chronic pain medication for other reasons, used prescription narcotics for concurrent acute conditions, had cognitive impairment that prevented them from understanding or answering questions, had failed to stop blood thinners, had significant perioperative chemosis or hematoma, were not contactable by telephone, or underwent unilateral surgery.

In Part II of the study, the aim was to determine the actual number of opioid pills used as well as use of any non‐narcotic opioid analgesics. Each patient undergoing surgery between November 2017 and January 2020 was prescribed 6 narcotic pills of either Norco or Percocet. At 2 weeks following surgery, patients were asked how many narcotic pills they had used and standardized questions regarding non‐narcotic analgesic use (“Did you use other over‐the‐counter [OTC] medication like Tylenol, extra‐strength Tylenol or Ibuprofen besides the prescription pain medication in your postoperative period?” and “Did you use any type of medical marijuana in your postoperative period?”). Patients were excluded based on criteria in Table 1.

**TABLE 1:** Exclusion criteria.

Part I	Part II
‐ Taking chronic pain medications or other prescription pain medications for other conditions ‐ Cognitive impairments limiting their ability to understand and answer study questions ‐ Inability to comprehend instructions in English ‐ Failure to stop blood thinning medications ‐ Significant perioperative chemosis or hematoma ‐ Not contactable by telephone ‐ Patients undergoing unilateral blepharoplasty/ptosis repair surgery	‐ Undergoing any other procedures at the same time ‐ Refusal of narcotics ‐ Taking chronic pain medications or other prescription pain medications for other conditions ‐ Known history of drug addiction or alcohol abuse ‐ Undergoing any other procedures within 2 weeks of surgery ‐ History of periorbital conditions that may require pain medication (i.e., uveitis, recurrent erosion syndrome, and floppy eyelid syndrome) ‐ Cognitive impairments limiting their ability to understand and answer study questions ‐ Inability to comprehend instructions in English ‐ Failure to stop blood thinning medications ‐ Significant perioperative chemosis or hematoma ‐ Not contactable by telephone

## 3. Results

**FIGURE 1:**
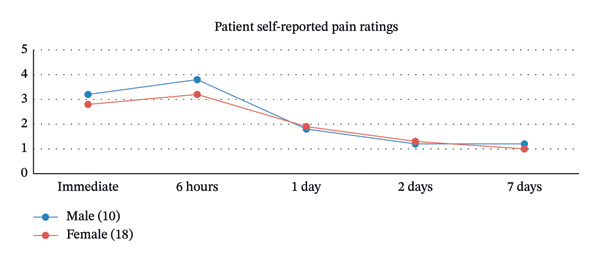
Patient self‐reported pain ratings.

In Part I of the study, patients were asked to rate their pain according to the standard 10‐point pain scale at various time points after surgery. Pain levels were higher in men and peaked at 6 h after surgery with an average rating of 3.8 and steadily decreasing to an average of 1.1 at 7 days postprocedure (see Figure 1).

In Part II of the study, there were 430 eligible patients undergoing bilateral upper eyelid surgery during the study period. 62 patients were excluded based on the criteria in Table 1 which left 368 patients who received 6 narcotic pills following blepharoplasty and/or ptosis surgery. Complete information was obtained from 286 patients, 108 male and 178 female. Among all patients, the mean number of narcotic pills used was 4.1. On average, male patients used slightly more pills than female patients (median 4.00 vs. 5.00, *p* < 0.001) as shown in Table 2. Five patients requested additional narcotic medication, and each was asked to come into the clinic for examination prior to receiving additional pain medication. Three chose to come in and had minimal pain on assessment and did not need further pain prescription medication, while the other two patients decided they did not need additional pain medication when asked to come in for evaluation.

**TABLE 2:** Postoperative opioid use (patient‐reported).

Characteristic	N	F, *N* = 178^1^	M, *N* = 108^1^	*p* value^2^
Age	286	65 (51, 76)	67 (57, 77)	0.072
No of pills	286	4.00 (2.00, 5.00)	5.00 (4.00, 6.00)	< 0.001

^1^Median (IQR).

^2^Welch Two Sample *t*‐test; Wilcoxon rank sum test.

**FIGURE 2:**
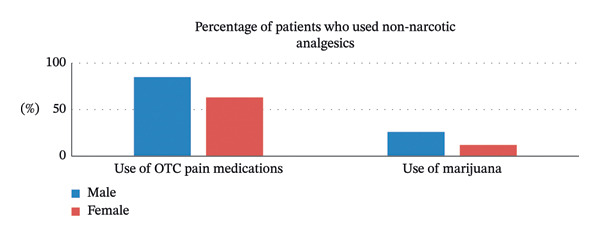
Percentage of patients who used non‐narcotic analgesics.

In response to the question “Did you use other OTC medications like Tylenol, extra‐strength Tylenol, or Ibuprofen besides the prescription pain medication in your postoperative period,” 205 of 286 participants (72%) answered that they used a non‐narcotic analgesic in their postoperative period (Figure 2). A higher percentage of male patients (85%) compared to female patients (63%) indicated use of non‐narcotic analgesics. When asked about use of medical marijuana in their postoperative period, 49 of 286 participants (17%) indicated use, with numbers higher in men (26%) than women (12%).

## 4. Discussion

The results of our study highlight several important points that warrant further discussion. First, we have established that blepharoplasty and ptosis repair have low levels of postoperative pain. Patients reported a maximum average rating of 3.4 at 6 h after surgery with pain levels falling steadily thereafter. Actual patient comments following surgery included “I was surprised by how little it hurt the morning after surgery,” and “I was swollen and looked like it should hurt, but it didn’t.” These values are similar to smaller published studies [8, 9] and give the surgeon confidence that postoperative pain should be mild and can be well‐controlled with reduced levels of opioids.

Second, we have established that 6 postoperative narcotic pills for blepharoplasty and ptosis surgery is a reasonable prescribing guideline. Our data shows that the mean number of pills used was 4.1, similar to smaller published results [10]. Additionally, only 5 patients requested additional pain medication, none of whom actually needed any after further clarification of their symptoms. Management of postoperative pain is a delicate balance between ensuring patients are comfortable and responsibly prescribing pain medication. Recently updated opioid prescribing recommendations from the CDC address this fact, with recommendations stating “clinicians should prescribe no greater quantity than needed for the expected duration of pain severe enough to require opioids.” [11] The number of 6 pills provides appropriate pain relief during the acute period after surgery when pain levels are highest without overprescribing leading to excess pills in circulation.

The majority of patients used non‐narcotic analgesic medications which likely reduced their need for opioids. Additionally, marijuana was used by 17% of patients, and its use will likely increase as legalization becomes more widespread. Additional research will be needed to determine the safety and efficacy of marijuana use as an adjunct for postoperative pain control.

As overdose deaths due to opioids continue to increase, responsible and thoughtful prescribing of opioids is more important now than ever before. There are multiple studies demonstrating that postoperative opioid prescriptions lead to an increase in long‐term opioid use and dependence, with one study reporting that patients who received opioids in cataract surgery were 1.6 times more likely to use opioids long‐term [12–14]. Additionally, most patients are overprescribed opioids, with 67%–92% of patients reporting having unused postoperative opioids [15]. This increases the risk for abuse not only in the patient, but in family members and friends, as up to 75% of heroin users state that they were introduced to opioids through prescription drugs [16].

Our study shows that by establishing the prescribing guideline of 6 postoperative opioid pills, opioid prescriptions can be safely reduced without compromising patient comfort and pain. By reducing the number of opioids prescribed from 15 pills per patient to 6 pills, this resulted in a reduction of over 1600 opioids prescribed in our population. Looking beyond our population, procedural statistics reporting from the Aesthetic Plastic Surgery National Databank indicates that nearly 150,000 blepharoplasty operations were performed in 2021 [17]. Extrapolating the results from our study would result in over 1.3 million fewer opioid pills prescribed. Standardizing prescriber guidelines has been shown to be effective in reducing opioid consumption. Specifically within ophthalmology, patients who underwent cornea surgery with prescribing guidelines in place were prescribed fewer opioids in the postoperative period [18].

This is an important reduction, but additional work needs to be done to continue to reduce opioid prescriptions further. As a group, ophthalmologists prescribe opioids at low rates, with the median prescriber rate at 4%, compared to the national average of 6.8% [19]. A study examining prescriber rates among members of the American Society of Ophthalmic Plastic and Reconstructive Surgery (ASOPRS) found prescribing rates of 16.5%, which were higher than the national average, but still half the rate compared to other surgical specialties [20]. Opioids are prescribed at higher rates in the United States compared to internationally [21], though there are ongoing efforts to move toward opioid‐free eyelid surgery. A recent publication from the Mayo Clinic stratified ophthalmic procedures into tiers based on surgeon expectations of postoperative pain, placing blepharoplasty into the “Level 0” tier, in which postoperative opioids were not recommended. After implementing this tiered system, there were significant reductions in both the number of opioid prescriptions issued and the quantity of pills prescribed without an increase in the rate of prescription refills [22]. Similar studies for other commonly performed procedures in oculoplastic surgery, like dacryocystorhinostomy, ectropion repair, entropion repair, and others, would help further reduce the prescription of unnecessary opioid medication.

Our study has several strengths and limitations. We have to date the largest sample size for a study examining opioid prescribing in oculofacial surgery for a commonly performed procedure. All patients underwent surgery with one surgeon with a consistent operative and postoperative routine. There were also robust exclusion criteria to minimize confounding variables. The main limitation of our study was the self‐reporting of opioid usage. Due to physical and logistical constraints, physically confirming each patient’s remaining opioid pills was not feasible. Our postoperative survey of patients is subject to the pitfalls inherent in any survey, including recall bias and failure to accurately report due to perceived expectations of the examiner.

In the midst of the worsening opioid epidemic, the results of our study demonstrate that opioid prescriptions can be safely lowered without adversely affecting patient outcomes. Establishing the parameter of 6 opioid pills after blepharoplasty and ptosis repair surgery provides surgeons an evidence‐based guideline to follow. Additional research will be needed to determine whether further reductions in opioid prescribing can be sustainably implemented, certainly a worthwhile goal in the effort to curb opioid abuse.

## Funding

This study was supported in part by an Unrestricted Grant from Research to Prevent Blindness, New York, NY, to the Department of Ophthalmology and Visual Sciences, University of Utah.

## Conflicts of Interest

The authors declare no conflicts of interest.

## Data Availability

The data that support the findings of this study are available on request from the corresponding author. The data are not publicly available due to privacy or ethical restrictions.
